# The Impact of Uric Acid-Lowering Therapy on the Progression of Non-dialysis Chronic Kidney Disease: A Prospective Cohort Study

**DOI:** 10.7759/cureus.70435

**Published:** 2024-09-29

**Authors:** Nghia N Nguyen, Tan Ngoc H Mai, Bao T Nguyen, Thuy Diem T Nguyen, Tam Thanh T Tran

**Affiliations:** 1 Faculty of Medicine, Can Tho University of Medicine and Pharmacy, Can Tho, VNM; 2 Medicine/Internal Medicine, Can Tho University of Medicine and Pharmacy, Can Tho, VNM

**Keywords:** chronic kidney disease, egfr, hyperuricemia, non-dialysis, serum uric acid

## Abstract

Background

Hyperuricemia treatment can positively influence the progression of chronic kidney disease (CKD). This study aimed to evaluate the impact of uric acid-lowering therapy on the progression of CKD after three months.

Materials and methods

A prospective cohort study was conducted on 126 patients with non-dialysis CKD in Can Tho Central General Hospital, Vietnam, from December 2018 to December 2019. Adopting a questionnaire survey method to collect information, including demographic characteristics, body mass index (BMI), personal history, previous medical history, diet, and use of drugs with the potential to affect the uric acid levels in the blood. Participants also underwent necessary tests, such as serum uric acid, serum creatinine, and blood lipids. Data were analyzed using SPSS software version 26.0 (IBM Corp., Armonk, NY).

Results

The prevalence of hyperuricemia in patients with non-dialysis CKD was 77.8%, and the average serum uric acid was 494.21 ± 131.57 µmol/L. Patients with a high-purine diet were about 18.85 times as likely to have hyperuricemia as those with a low-purine diet (p < 0.001, OR = 18.85, 95%CI: 4.233-83.938). BMI, stages of CKD, and hypertension were associated with the hyperuricemia rate (p < 0.05). After three months of treatment, 46.2% of patients achieved the serum uric acid target, and the patient group combined allopurinol with changing diet had a higher rate than the patient group changing diet only (p = 0.02). There was a moderate inverse correlation between the difference in serum uric acid and the difference in estimated glomerular filtration rate (eGFR) after treatment (r = -0.5, p = 0.001).

Conclusions

The effective management of hyperuricemia by combining nonpharmacological (changing diet) and/or pharmacological (allopurinol) therapies may meaningfully improve the glomerular filtration rate in non-dialysis CKD patients with hyperuricemia.

## Introduction

Chronic kidney disease (CKD) is an increasing global public health issue [1], with an estimated overall prevalence of 8%-16% [2]. Nearly 500 million individuals are affected by this condition, with 78% (approximately 387.5 million) residing in low- to middle-income countries. Between 1990 and 2010, deaths attributed to CKD almost doubled, making it the 18th leading cause of death in 2012. Although the exact burden of CKD in low- to middle-income countries has yet to be fully understood, it is estimated that the incidence rates may be up to four times higher than those observed in developed countries [3]. The expenditures of treatment and non-medical activities (travel for medical appointments, time off from work) impact the national economy directly and indirectly [4]. Furthermore, CKD patients also need support from relatives and friends because the psychological factors of illness and disease treatment severely affect the quality of life [5].

Many factors influence the severity of CKD, including hypertension, diabetes, hyperlipidemia, hyperuricemia, and others. An imbalance between synthesis and excretion causes hyperuricemia. Uric acid is both a marker and a risk factor for kidney failure [6,7]. Deposing urate crystals in the kidney causes nephrolithiasis and urate nephropathy [8]. Studies suggest a correlation between the prevalence of hyperuricemia and CKD [9]. Hyperuricemia treatment effectiveness potentially slows CKD progression [10-12]. Treatment options include lifestyle changes, diet control, increased physical activity, and medication. Specifically, allopurinol is the recommended first-line treatment to manage high uric acid concentration in the blood.

In Vietnam, the age of end-stage kidney disease patients is relatively young [13]. Therefore, slowing the progression of CKD by controlling risk factors and underlying causes such as hyperuricemia is extremely important, especially when the disease is in its early stages and regular dialysis is not yet required. However, there have not been many studies on hyperuricemia in CKD patients in Vietnam, the diagnosis, management, and prevention of hyperuricemia-related kidney disease are ineffective. The present study aims to assess the impact of hyperuricemia management on the progression of non-dialysis CKD.

## Materials and methods

Study design

This study was a prospective cohort conducted on patients with CKD at Can Tho Central General Hospital, Vietnam, from December 2018 to December 2019. Patients ≥ 18 years old with non-dialysis CKD (K/DOQI 2002) were included in the study [14]. Patients used the following drugs: allopurinol, febuxostat, probenecid, sulfinpyrazone, salicylate, ethambutol, thiazide, furosemide, and pyrazinamide (because of the effects of these medications on the synthesis and excretion of uric acid) were excluded from the study. In addition, patients with underlying medical conditions were excluded, including acute gout attacks, acute exacerbation of chronic renal failure, aphasia, or mental illness, since these diseases had impacts on the objectivity of the results and the sample collection process. The study adhered to the principles of the Declaration of Helsinki and received approval from the Ethics Committee of Can Tho University of Medicine and Pharmacy (approval no. 2661/QD-ĐHYDCT dated December 27, 2019). Informed consent was obtained from all participants involved.

A standardized questionnaire was used to collect demographic and clinical data of patients who met the entry criteria. The general characteristics included age, gender, body mass index (BMI), alcohol consumption, smoking, high-purine diet, history of any illnesses (hypertension, diabetes, chronic gout, dyslipidemia), and CKD stage. Then, the participants carried out serum uric acid and other tests to determine the status of hyperuricemia. Patients with hyperuricemia who agreed to continue the study were transferred to phase 2 to receive treatment (in three months). Patients were advised to change their diet if serum uric acid concentration ≥ 540 μmol/L and/or complications of hyperuricemia such as patients with joint symptoms (gout) and/or high cardiovascular risk factors (hypertension, diabetes), they would be treated with allopurinol with a starting dose of 100 mg/day, administered orally, once a day after meals; adjust increments up to 300 mg/day (depending on the patient). Other comorbidities are treated (if any).

**Figure 1:**
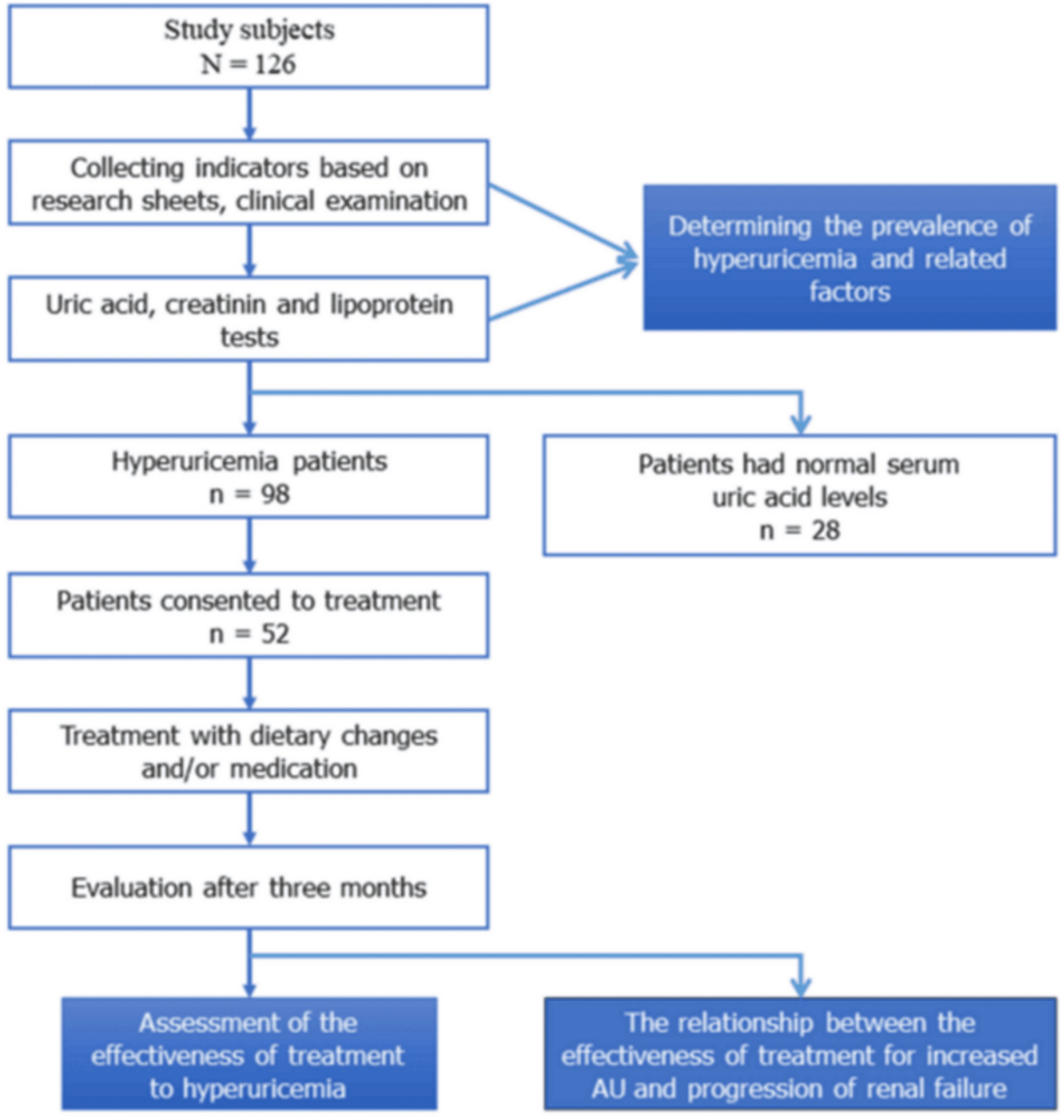
Flow chart showing sample size collection and objectives of the study

The serum uric acid concentration target was 360 μmol/L for women and 420 μmol/L for men. The progression of CKD was considered slow or reached the target if the estimated glomerular filtration rate (eGFR) after three months compared with that of the initial was less than 0.6 mL/min/1.73 m^2^. Figure 1 depicts the sampling and objectives of the study.

Data collection

Hyperuricemia was diagnosed when the uric acid concentration was > 420 μmol/L in men and > 360 μmol/L in women [15], including three levels [16]: mild (normal to 550 μmol/L), moderate (550-900 μmol/L), severe (> 900 μmol/L). Hypertension was defined as blood pressure > 140/90 mmHg according to JNC VII. Diabetes was defined according to the guidelines of ADA 2017. Chronic gout was defined according to the guidelines of ACR/EULAR 2015. Dyslipidemia was defined according to the guidelines of NCEP ATP III. Patients were divided into five stages based on their glomerular filtration rate (GFR) assessed using the 2009 CKD-EPI method (according to KDOQI 2002). Body weight was measured using an electronic balance with indoor clothing without shoes. Height was measured on a handheld stadiometer without shoes. BMI, derived as the weight in kilograms divided by the square of the height in meters, is classified according to WPRO 2000 [17]. Alcohol consumption was defined as ≥ 2 and ≥ 1 standard drinks in men and women, respectively. No smoking if the patient never smoked or stopped smoking for more than 12 months [18].

Statistical analysis

We analyzed the data using Statistical Package for the Social Sciences (SPSS) version 26.0 (IBM Corp., Armonk, NY). Qualitative variables were described by frequency and percentage, and quantitative variables were presented as mean ± standard deviation. Compare the relationship between two qualitative variables by testing (Chi-squared) and between two quantitative variables by t-test with 95% confidence. Determine the correlation coefficients of two quantitative variables: the Pearson correlation coefficient if the variables were normally distributed and the Spearman correlation coefficient if the variables were a non-normal distribution (positive r: positive correlation, negative r: negative correlation). The degree of correlation (|r|) was determined (|r| < 0.3: weak correlation, |0.3| ≤ r < |0.7|: moderate correlation, |r| ≥ 0.7: strong correlation). The difference was statistically significant when p < 0.05.

## Results

The research was conducted on 126 non-dialysis CKD patients. Among them, 61.1% were male; the mean age was 69.1 ± 16.65. The average BMI was 22.5 ± 2.99 kg/m^2^ (Table 1). Factors associated with hyperuricemia in non-dialysis patients included BMI levels, a high-purine diet, hypertension, and CKD stages. Patients who ate a high-purine diet were about 18.85 times as likely to have hyperuricemia as people who ate the low-purine diet (p < 0.001).

**Table 1:** Patient characteristics and serum uric acid status (n = 126) CKD = chronic kidney disease; BMI = body mass index; SD = standard deviation; OR = odds ratio. Statistical significance: p < 0.05.

Characteristics		Total, n (%)	Serum uric acid status		P-value
			Increased, n (%)	Not increased, n (%)	
Age	< 60	32 (25.4)	26 (81.3)	6 (18.8)	0.58
	≥ 60	94 (74.6)	72 (76.6)	22 (23.4)	
	Age (mean ± SD): 69.1 ± 16.65				
Gender	Male	77 (61.1)	56 (72.7)	21 (27.3)	0.087
	Female	49 (38.9)	42 (85.7)	7 (14.3)	
BMI	Underweight	15 (11.9)	8 (53.3)	7 (46.7)	0.02
	Normal	63 (50)	48 (76.2)	15 (23.8)	
	Overweight	48 (38.1)	42 (87.5)	6 (12.5)	
	BMI (mean ± SD): 22.5 ± 2.99 kg/m^2^				
Alcohol consumption	Yes	51 (40.5)	41 (80.4)	10 (19.6)	0.56
	No	75 (59.5)	57 (76)	18 (24)	
Smoking	Yes	41 (32.5)	31 (75.6)	10 (24.4)	0.68
	No	85 (67.5)	67 (78.8)	18 (21.2)	
Hypertension	Yes	110 (87.3)	91 (82.7)	19 (17.3)	0.002
	No	16 (12.7)	7 (43.8)	9 (56.3)	
Diabetes	Yes	33 (26.2)	28 (84.8)	5 (15.2)	0.255
	No	93 (73.8)	70 (75.3)	23 (24.7)	
Chronic gout	Yes	9 (7.1)	9 (100)	0 (0)	0.2
	No	117 (92.9)	88 (76.1)	25 (23.9)	
High-purine diet	More often	60 (47.6)	58 (96.7)	2 (3.3)	0.001 (OR = 18.85)
	Less often	66 (52.4)	40 (60.6)	26 (39.4)	
CKD stage	Stage 1	1 (0.8)	8 (53.3)	7 (46.7)	0.047
	Stage 2	14 (11.1)			
	Stage 3	58 (46)	46 (79.3)	12 (20.7)	
	Stage 4	53 (42.1)	44 (83)	9 (17)	

The proportion and level of hyperuricemia in participants are shown in Table 2. There were 77.8% of CKD patients had hyperuricemia, and the majority were in mild and moderate hyperuricemia levels.

**Table 2:** Prevalence and level of hyperuricemia (n = 126) SD = standard deviation.

Characteristics		Number of patients	Percent
Serum uric acid status	Not increased	28	22.2
	Increased	98	77.8
Hyperuricemia levels	Mild	64	65.31
	Moderate	33	33.67
	Severe	1	1.02
Serum uric acid (mean ± SD)		494.21 ± 131.57 µmol/L	

Serum uric acid and factors associated with hyperuricemia in non-dialysis patients are shown in Table 3. The serum uric acid was the lowest in stage 1 and stage 2 CKD groups and increased according to the CKD stage.

**Table 3:** Serum uric acid and factors associated with hyperuricemia CKD = chronic kidney disease; BMI = body mass index; SD = standard deviation. Statistical significance: p < 0.05.

Factors		Serum uric acid (µmol/L), mean ± SD	P-value
BMI levels	Underweight	410.73 ± 124.29	0.001
	Normal	427.71 ± 102.21	
	Overweight	548.50 ± 147.65	
Hypertension	Yes	507.28 ± 130.17	0.003
	No	404.31 ± 106.1	
High-purine diet	More often	574.65 ± 124.14	0.001
	Less often	421.08 ± 88.86	
CKD stage	Stages 1, 2	412.87 ± 80.37	0.032
	Stages 3	496.66 ± 121.87	
	Stages 4	512.36 ± 146.03	

Fifty-two patients agreed to treat hyperuricemia by diet changes and/or taking medication. After three months, the percentage of patients achieving the serum uric acid target was 46.2%. Up to 60.7% of them were treated by combination diet change with allopurinol (p = 0.02) (Table 4). The majority of patients returned to normal or mild hyperuricemia, and the mean of serum uric acid decreased by about 88.92 ± 168.91 µmol/L compared to before treatment (p = 0.001) (Table 5).

**Table 4:** Effectiveness of treating hyperuricemia and treatment measures (n = 52) Statistical significance: p < 0.05.

Serum uric acid target	Total, n (%)	Diet change, n (%)	Diet change + allopurinol, n (%)	P-value
Yes	24 (46.2)	7 (29.2)	17 (60.7)	0.02
No	28 (53.8)	17 (70.8)	11 (39.3)	

**Table 5:** Hyperuricemia levels and serum uric acid before and after treatment (n = 52) SD = standard deviation. Statistical significance: p < 0.05.

Hyperuricemia levels	Before, n (%)	After, n (%)	P-value
Normal	0 (0)	24 (46.2)	-
Mild	38 (73.1)	19 (36.5)	
Moderate	13 (25.0)	9 (17.3)	
Severe	1 (1.9)	0 (0)	
Serum uric acid (µmol/L) (mean ± SD)	522.3 ± 114.6	433.4 ± 133.9	0.001

The effectiveness of treating hyperuricemia and the progression of CKD is shown in Tables 6-8. After three months, the percentage of patients achieving the eGFR garget was 55.8% (Table 6), and eGFR was higher than before treatment, about 1.67 ± 5.9 mL/min/1.73 m^2^ (p = 0.047) (Table 7). After treatment, 70.8% of patients who achieved the serum uric acid target reached the eGFR target (p = 0.04) (Table 8).

**Table 6:** Rate of patients achieved the eGFR goal (n = 52) eGFR = estimated glomerular filtration rate.

eGFR goal	Number of patients	Percent
Yes	29	55.8
No	23	44.2

**Table 7:** eGFR before and after treatment (n = 52) eGFR = estimated glomerular filtration rate; SD = standard deviation.

	eGFR (mL/min/1.73 m^2^), mean ± SD	P-value
Before	32.42 ± 12.84	0.047
After	34.09 ± 15.63	

**Table 8:** Relationship between serum uric acid and eGFR goals after three months of treatment (n = 52) eGFR = estimated glomerular filtration rate

eGFR goal	Serum uric acid goal		P-value
	Yes, n (%)	No, n (%)	
Yes	17 (70.8)	12 (42.9)	0.04
No	7 (29.2)	16 (57.1)	

**Figure 2:**
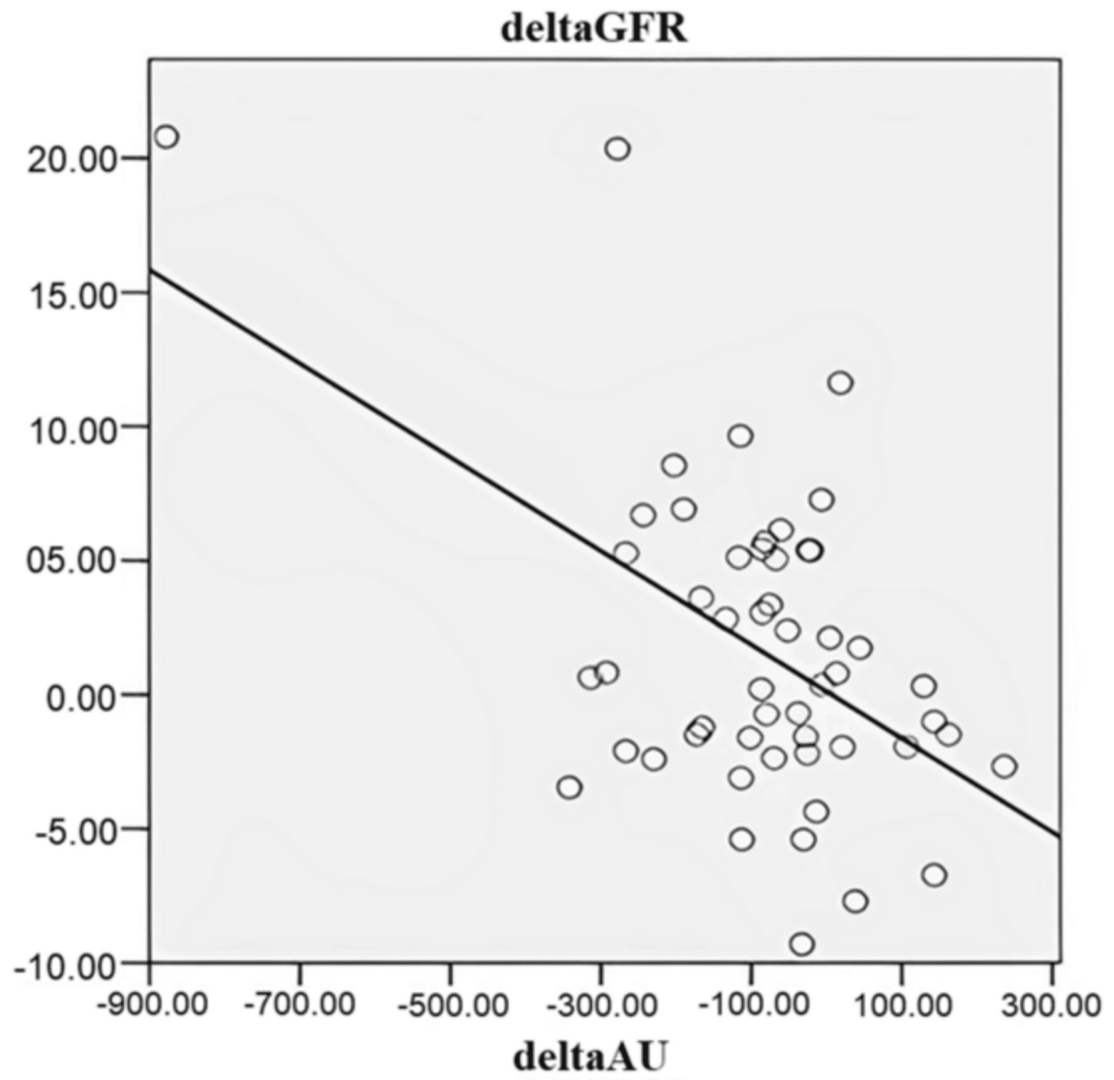
Correlation between the difference in serum uric acid and the difference in eGFR eGFR = estimated glomerular filtration rate

The correlation between the difference in serum uric acid and the difference in eGFR after three months of treatment is shown in Figure 2. There was a moderate inverse correlation between the difference in serum uric acid and the difference in eGFR after three months of treatment (r = -0.5, p = 0.001).

## Discussion

There were 77.8% of CKD patients with hyperuricemia, and most of them were at a mild level. This result was similar to the recent study by Le et al. (74.4%) [17]. The mean of serum uric acid was consistent with studies of Pan [7] and Huynh et al. [19].

Overweight patients had a higher risk of serum uric acid status than others (p = 0.02), and the higher the BMI levels of patients were, the higher the serum uric acid they had (p = 0.001). This result is reinforced by previous documents [20,21]. In overweight patients, decreased uric acid secretion and increased uric acid production cause higher serum uric acid. Besides, the rate of hyperuricemia in patients who ate a high-purine diet more often was higher than in the other group (p = 0.001). The risk of hyperuricemia was 18.85 times higher (OR = 18.85; p = 0.001). The diet could increase the exogenous purines, which break down into uric acid and cause high uric acid levels. The finding has also been observed in several studies [22,23]. Our study found that the prevalence and level of hyperuricemia in patients with hypertension were higher than in patients without hypertension (p < 0.01), similar to other reports [16,18,20]. We also observed that the serum uric acid increased according to CKD stages (p < 0.05). Tsai et al. reported that higher uric acid levels are associated with a significantly more rapid decline in eGFR and a higher risk of kidney failure, particularly in patients without proteinuria. Hyperuricemia is a modifiable factor in the progression of CKD [24]. Le et al. also indicated that the proportion of patients with hyperuricemia was proportional to the stage of CKD [18]. Similar findings were also found by Huynh et al. [19]. Almost all uric acid is filtered from glomeruli, while post-glomerular reabsorption and secretion regulate the amount of uric acid excretion. In mild CKD patients, the secretion and absorption system of the kidney is still preserved, so hyperuricemia is not high; however, in severe renal failure, the secretion and absorption system are unstable, which leads to hyperuricemia.

There were no significant differences in gender, age, and alcohol consumption with the rate of hyperuricemia in the study, observations supported by previous data [14]. However, Li et al. concluded that male alcohol consumption had a 1.7 times higher risk of hyperuricemia than other groups [25]. Purine degradation during ethanol catabolism, inhibition of renal excretion of urate by lactic acid, and high purine content of certain alcohols are responsible for elevated serum uric acid levels following alcohol drinking [26]. In our study, most patients were older men, and the alcohol consumption rate was not high. Most female patients were over 60 years old - the age of post-menopause, and estrogen reduced, so renal function gradually decreased with age, leading to increased serum uric acid. There was no difference in the rate of hyperuricemia in smokers and non-smokers (p = 0.68); this result was similar to the research of Tran et al. [16]. The higher the patients' serum uric acid level, the higher their risk of gout. However, the gout patients in our study may have been low (9/126), so we did not record the relationship between serum uric acid and gout. There were no significant differences in hyperuricemia between patients with and without diabetes; this result was similar to Kuo’s research [27]. Many studies ascertained the relationship between hyperuricemia and dyslipidemia [18,20,25]. However, the difference was not statistically significant (p = 0.43).

Serum uric acid levels decreased after three months of treatment; 46.2% of patients had serum uric acid returning to normal. Most remaining patients had mild hyperuricemia levels, and none had severe hyperuricemia ones. Among 46.2% of patients who achieved the serum uric acid target, up to 60.7% were treated by combination diet change with allopurinol (p = 0.02). Some findings demonstrate that diet change helped reduce only about 1 mg/dL within one day. Therefore, diet change should be combined with medicine in hyperuricemia treatment [28]. Our study selected allopurinol instead of febuxostat because these drugs had similar side effects, especially febuxostat's cardiovascular events, which were higher than allopurinol [29].

We found that 100% of stage 1 and 2 CKD patients and 62.5% of stage 3 CKD patients achieved the serum uric acid target after 3 months of treatment. In contrast, most stage 4 CKD patients did not reach the target (73.1%). In normal humans, 2/3 of uric acid is excreted in the urine. Therefore, uric acid excretion may be impaired by kidney disease, leading to hyperuricemia. Allopurinol is an inhibitor of xanthine oxidoreductase and inhibits the generation of uric acid in humans. The effect of uric acid reduction was more significant when patients used it in increasing doses. For all patients initiating allopurinol, the starting dose should be low, specifically 50 mg/day for CKD stage 4 or 5, and no more than 100 mg/day in all others [28]. In patients with severe CKD, the kidneys filter uric acid from the blood and pass it out of the body in the urine weakly; allopurinol dosage is also limited; therefore, the treatment is ineffective. Some studies showed the opposite results. Tiku et al. argued that there was not enough evidence about the role of urate-lowering therapy in slowing the progression of CKD [29]. Compared to placebo, febuxostat did not mitigate the decline in kidney function among patients with stage 3 CKD and asymptomatic hyperuricemia [30]. This difference might be that their study was conducted in patients with CKD stage 3 and above. In addition, there were differences in the participants' ethnicity, culture, and educational background. This sets the stage for further studies on the characteristics of CKD patients and focuses on patients with CKD in the early stages.

Assessing the effectiveness of hyperuricemia treatment and the progression of CKD, we found that 70.8% of patients who achieved serum uric acid control reached the eGFR target (p = 0.04). There was a moderate inverse correlation between the difference in serum uric acid and the difference in eGFR after three months of treatment (r = -0.5, p = 0.001). That meant that when serum uric acid levels decreased, eGFR increased. Goicoechea [10] and Pai [12] also recorded similar results. In Goicoechea’s study, after 24 months, in the control group, eGFR decreased 3.3 ± 1.2 mL/min per 1.73 m^2^, and in the allopurinol group, eGFR increased 1.3 ± 1.3 mL/min per 1.73 m^2^, so that, allopurinol treatment slowed down renal disease progression independently of age, gender, diabetes, C-reactive protein, albuminuria, and renin-angiotensin system blockers use [10]. Besides, In a retrospective cohort study in CKD patients with hyperuricemia at Nizam's Institute of Medical Sciences, Hyderabad from 1998 to 2008, patients who received allopurinol had lower blood pressure at six months, one year, and two years when compared to baseline. There was a significant decrease in the serum uric acid levels in the treatment group at the end of six months, one year, and two years with respect to baseline. An inverse correlation was noted between serum uric acid levels and the eGFR at six months, one year, and two years [12]. In general, hyperuricemia is strongly associated with the progression of renal failure, and that treatment is beneficial in slowing this progression in CKD patients. The precise mechanism is still unclear, and hyperuricemia may rise in glomerular hydrostatic pressure, stimulating vascular smooth muscle cell proliferation, which is a critical event in the progression of arteriosclerosis in the kidney. Allopurinol treatment reduces serum uric acid levels, thus decreasing hydrostatic pressure in the glomerular capillary indirectly and helping to prevent further kidney damage [12].

This study is a prospective cohort to show clear cause-and-effect relationships. The study evaluated the effect of treating hyperuricemia on the progression of CKD and focused on the association between the difference between serum uric acid and eGFR after treatment. Besides, we chose allopurinol instead of febuxostat because of its reasonable price, so it was suitable for long-term treatment in Vietnam. In the study, we also found that most Vietnamese patients had mild to moderate elevation of serum uric acid, a few increased in severity. Moreover, patients with advanced CKD had higher uric acid levels. Therefore, we believed managing serum uric acid levels in patients with CKD from the early stages was necessary. Patients with severe hyperuricemia required more aggressive control, with multiple measures (e.g., medication in combination with lifestyle changes).

Limitations

The study also had some limitations. First, this study used a relatively small sample size (52 patients) to evaluate the role of urate-lowering agents in CKD progression in a short period of follow-up (three months). Therefore, it is necessary to conduct randomized controlled trial research to estimate the effect of urate-lowering agents use on renal function in a larger sample with longer follow-up time in the future. Second, confounding factors could not be excluded entirely since this study did not have information on other nephrotoxic or renoprotective agents (e.g., aminoglycosides or contrast); some blood pressure medications can have the added benefit of lowering uric acid (losartan, calcium channel blocker) and adherence to urate-lowering agents. Third, the usage of diuretics was not obtained during data collection. The use of diuretics may be linked to increased hyperuricemia. However, the association between diuretic use and eGFR decline is controversial. Therefore, our following studies will focus on patients with and without diuretics. Fourth, this study's etiologies of hyperuricemia were not systematically determined. Patients with primary or secondary hyperuricemia and hyperuricemia because of increased production or decreased excretion were not distinguished clearly, requiring further research. Finally, multivariate regression analysis should be considered in further studies.

## Conclusions

The study conducted on 126 non-dialysis CKD patients at a hospital in Can Tho, Vietnam, revealed that the prevalence of hyperuricemia was 77.8%, with most cases being mild to moderate. Factors such as BMI, a high-purine diet, hypertension, and CKD stages were closely associated with the occurrence of hyperuricemia. After three months of treatment, the effective management of hyperuricemia by combining nonpharmacological (changing diet) and/or pharmacological (allopurinol) therapies may meaningfully improve glomerular filtration rate in non-dialysis CKD patients with hyperuricemia.
